# Ukrainian refugee women’s experience with maternity care in Norway: A qualitative study

**DOI:** 10.18332/ejm/200613

**Published:** 2025-02-12

**Authors:** Mirjam Lukasse, Fatima Akhmedova, Hanna Oommen

**Affiliations:** 1 Department of Nursing and Health Sciences, University of South-Eastern Norway, Borre, Norway; 2 Department of Nursing and Health Promotion, Oslo Metropolitan University, Oslo, Norway; 3 Department of Obstetrics and Gynecology, Sykehuset Innlandet Lillehammer, Lillehammer, Norway; 4 Department of Obstetrics and Gynecology, Sorlandet Hospital Kristiansand, Kristiansand, Norway

**Keywords:** childbirth, refugee, migrant, Norway, respectful care, cultural care

## Abstract

**INTRODUCTION:**

European countries have recently received many migrants from Ukraine. Women’s life experiences and expectations shape their perception of maternity care and childbirth. Our study aimed to explore how newly arrived Ukrainian refugee women experience their maternity care in Norway.

**METHODS:**

Social media were used to recruit eight women meeting the criteria of being newly arrived Ukrainian refugee women. Semi-structured interviews, three face-to-face and five video-calls, were performed between December 2023 and January 2024. We used Braun and Clarke for thematic analysis.

**RESULTS:**

Three main themes were identified: healthcare in country of origin, high-quality care in the new country, and challenges as a refugee. Women reported that in their home country, the cost of maternity care had a significant impact on the quality of care they received with a tendency toward overmedicalization, as access to certain services often depended on payment. Participants described instances of feeling disrespected by healthcare staff in their home country, in contrast to their experiences in Norway. Women reported that in Norway they experienced a high level of professionalism among healthcare staff and a well-functioning healthcare system with good physical conditions. Challenges that the women struggled with were communication and barriers to accessing services.

**CONCLUSIONS:**

Being treated professionally and with respect played a central role in creating a positive experience and mitigated the negative experiences of problems with communication and navigation in an unfamiliar healthcare system. Future research could investigate the use of written information to aid migrants in understanding the maternity services and some of the vocabulary.

## INTRODUCTION

Since February 2022, most European Union countries have received a substantial number of Ukrainian refugees^1^. In Norway, most Ukrainian refugees have been included in a collective protection scheme involving a rapid process, favorable economic benefits, and the intention of return to their home country^2^. By January 2024, about 25000 adult women were living in Norway under this scheme^2^. Ukrainians were then the largest group of new migrants to Norway, closely followed by Syrian and Somalian migrants^2^.

Research on maternal and neonatal outcomes for migrants giving birth in high-income host countries is equivocal^3^. Some research suggest that migrants have poorer health outcomes than those born in the host country, while others highlight the inconsistent findings and call for more research^3,4^. Major contributors to poorer outcomes are challenges in accessing care, navigating healthcare systems, communication problems, and mental health issues^5,6^.

Ukrainian migrants share the traumatic experience of migration due to conflict with many other migrants^6,7^. Their socio-economic and cultural background, however, is quite different from other recent migrants to Norway, such as Syrian and Somalian refugees^8^. Ukraine is a nation defending its national sovereignty, following Europe’s historical trajectory of globalization, modernization and increased wealth^9^. A large population-based study found that migrant women from countries with a higher Human Development Index score (HDI score) had similar or better outcomes compared to non-migrant women and migrant women from countries with a low HDI score^10^.

One maternal outcome which is receiving an increasing interest is the experience of childbirth^11^. The importance of a positive childbirth experience is apparent by the inclusion in the aims of the WHO guidelines for childbirth care^12^. According to the Quality Maternal Neonatal Care Framework, this requires care tailored to women’s circumstances and needs^13^.

A systematic review on migrant women’s experiences of pregnancy, childbirth and maternity care in European countries found that migrant women need culturally competent healthcare providers to meet their needs^14^.

We found little research investigating Ukrainian women’s experiences with maternity services in Europe^15,16^. One qualitative study with Polish midwives reports that Ukrainian women shared many traumatic experiences, suggesting an increased need for emotional care^16^. The aim of our study was to explore how newly arrived refugee women from Ukraine experienced maternity care in their new host country, Norway.

## METHODS

We performed a qualitative thematic study, collecting data through semi-structured interviews of eight Ukrainian refugee women from all over Norway between December 2023 and January 2024.

All three authors migrated to Norway as adults and experienced language and cultural barriers themselves. The similar experiences to the participants in the study assisted in obtaining the women’s trust and openness when interviewed as well as the authors’ understanding of the women’s background.

The study was announced in Russian on social media platforms used by and relevant to Ukrainian women at the start of December 2023. Russian is either the primary or secondary language for most Ukrainian citizens. The Russian speaking author’s contact details were in the announcement. Inclusion criteria were being a Ukrainian refugee fluent in Russian, having arrived since February 2022, and having given birth in Norway. As recruitment was through social media with the opportunity of digital participation, women from all over Norway could participate. Initially, 16 women showed an interest in the study by contacting the first author via text message. One potential participant declined further participation after learning more about the study.

The interview guide started with one open question about their childbirth experience in Norway and was followed up with four to six planned questions. Minor revisions were made in the interview guide after the first two interviews (Supplementary file).

The interviews were performed in the place/format of choice by the women. Three were at the homes of the women and five were through a video-call. They lasted on average 40 minutes, the shortest lasting 24 minutes and the longest 67 minutes. After the interview of participant number eight, there were a total of 80 pages of transcription and it was decided that sufficient data had been collected to be able to meet the aim of our study. There were seven more women who had shown an interest in participating, but they were informed that sufficient information was obtained, and interviewing them as well was not necessary. The final sample consisted of eight women.

Ethical approval was not required. As part of the ethical considerations a contingency plan for referral to other services was put in place. At the start of the interview, women received verbal and written information about how their privacy was being protected in accordance with the Norwegian law governing medical research^17^. Women were informed that they could withdraw from the interview at any time and request to be removed from the study as long as their data had not been incorporated with other data. The interviewer was aware that the interview could be emotionally disturbing and offered to pause the interview if needed. Women provided written consent. During the interview with Participant 2, it was revealed that she had given birth in Norway while visiting her boyfriend two years before arriving as a refugee. After careful consideration, it was determined that she still met the selection criteria, despite this unexpected situation.

The study was submitted and evaluated by Norway’s governmental institution guarding the collection and storage of data and person privacy (Sikt nr.187993) in November 2023. Recordings were de-identified using a key/number to link the interview to background information. Background information was collected separately from the recording. The interviews were recorded via an app (Diktafon) and stored in secure cloud services owned by the University of Oslo (Nettskjema.no).

### Analysis

Data was analyzed using the Braun and Clarke^18^ method for thematic analysis. We aimed at inductive analyses, allowing themes and subthemes to be determined by the data. The first step in this analysis is getting to know the data through transcription, repeated reading and reflection. Each interview was transcribed and translated by the Russian speaking author. The transcriptions of the first two interviews were read and reflected upon prior to the subsequent interview. To validate the translation half of the interviews were listened to and their text read by a Ukrainian obstetrician fluent in Russian. Minor changes were made to the transcriptions. The next step is finding and coding text extracts that are of interest. Approximately 100 different codes were identified. The third step in the Braun and Clarke^18^ method is identifying themes and sorting codes under themes. Fourthly, subthemes are identified and organized under main themes. Finally, the main themes and subthemes are labelled. The analysis process is iterative. Themes and subthemes are revisited, overlap is avoided, names/labels are reworded until the themes and subthemes appear to present the findings in a structured, understandable way, being true to what women said. The final step described by Braun and Clarke^18^ is writing the results which are given in the next section.

## RESULTS

The eight participants in our study were aged 25–38 years, four of them had a college/university education and had been in Norway between 2 and 20 months (Table 1). They had varying parity and method of childbirth (Table 1). Three main themes were identified, each with two subthemes as shown in Table 2.

**Table 1:** Characteristics of the eight participants in the qualitative study on Ukrainian women’s experiences of maternity care in Norway, December 2023 – January 2024

*Participant number*	*Age (years)*	*Education level*	*Length of stay in Norway (months)*	*Method of childbirth*	*Number of months since birth*	*Support person present besides medical staff*	*Use of aid for communication*
1	25	High school	2	Emergency cesarian section	1	None	Digital translate
2	36	College/University	18	Emergency cesarian section	12	Partner	Partner
3	27	College/University	14	Spontaneous vaginal birth	42	None	Digital translate
4	35	High school	7	Spontaneous vaginal birth	1	None	English
5	38	College/University	10	Spontaneous vaginal birth	1.5	None	Doula via telephone
6	34	High school	7	Spontaneous vaginal birth	1.5	Daughter	English with daughter
7	36	College/University	20	Elective cesarian section	11	Social services person	Social services person
8	26	High school	18	Vacuum extraction	1.5	Partner	Interpreter on phone/partner

**Table 2:** Themes and subthemes in the qualitative study on Ukrainian refugee women’s experiences with maternity care in Norway, December 2023 – January 2024

*Themes*	*Subthemes*
**Healthcare in country of origin**	Financial aspects of healthcare
	Lack of respect
**High-quality care in new country**	Highly professional and caring staff
	Good healthcare system and facilities
**Challenges of being a refugee**	Problems communicating
	Barries to accessing services

### Healthcare in country of origin

All participating women, also those who had not given birth in Ukraine, had knowledge of the healthcare system in their country and wanted to share this. Participants who had given birth in Ukraine, pointed out how this previous experience influenced their expectations.

### Financial aspects of healthcare

All participants mentioned that in contrast to free healthcare in Norway, the cost of care was a big issue in their home country. One participant said that you start saving up money before or as soon as you are pregnant. Participants suggested that healthcare staff in their country were highly motivated by economic gain in their practice. Women believed that both the quantity and quality of treatment and care were influenced by the opportunity to increase economic gain leading to overmedicalization, including unnecessary investigations and interventions. Several women said that if they had given birth at home, they would very likely have ended up doing so by cesarian section:

*‘… and our doctors earn a lot of money on that [interventions], because our healthcare is not free. Nothing is free. So, they try and pump for as much as possible out of her in this period.’* (Participant 7)

Participants emphasized that not only the content of the medical treatment but also the level and quality of care depended on the economic status of the patient. Several women mentioned a lack of care in the public health services compared to the care available in the private sector. As one participant expressed it:

*‘In Ukraine, if you go to a private clinic, they will treat you like a goddess.’* (Participant 5)

When in public care it is possible and even necessary to pay for added services. Several women explained that pain relief in labor was not automatically included but could be arranged upon extra payment and separate agreements with doctors:

*‘Because in Ukraine, when you give birth, you have a contract with an anesthesiologist. You pay him separately and pay for the medicines. And you can choose that medication. He tells you this is more expensive, and this is cheaper, and what you want.’* (Participant 7)

Paying for services was not viewed as solely negative. Several women reported that it allowed them to influence their care and make their own choices, as long as they had enough economic resources.

### Lack of respect

Women reported that maternity care in their country was characterized by lack of both respect and empathy. They described care that made patients feel worthless and dehumanized. Women with personal experience of maternity care reported being exposed to degrading behavior through both actions and words. They were made to feel that they were a burden to the staff. Midwives openly expressed irritation and were displeased. Several women reported that their basic needs were neglected:

*‘If you give birth in a public hospital, you are treated like you are in a factory. I mean, there are many women giving birth there. The staff are angry and tired. Everybody understands that. But you are treated like you are a burden.’* (Participant 5)

Women experienced that healthcare staff had no interest in them. They were not present, neither physically nor mentally. As one woman said:

*‘She [the midwife] sat at the other end of the room and talked on her phone with a guy. I said, “it feels like I am giving birth”. She sat there and said, “open your legs”. She said, “I cannot see anything. No”.’* (Participant 6)

Women’s basic needs were not prioritized and came second to what suited the staff best. Midwives were more concerned with reducing their workload than women’s comfort. Women reported not receiving anything to drink or eat to avoid extra work for staff in case they should vomit later. Women did point out that there is a relationship between paying staff money and respectful care. Extra payment increased respect, lack of payment decreased respect.

### High-quality care in Norway

Women were very satisfied with the healthcare system and staff in Norway. They constantly compared it to the healthcare system in their home country. Both staff and facilities were highly rated.

### Highly professional and caring staff

Women reported trusting the healthcare staff, not only midwives, as they felt confident in their expertise. Their actions and body language generated trust. They described how staff with different professional backgrounds worked well together as a team, complementing each other’s skills:

*‘It all went very quickly in a way. They worked like a clock. There were 8 people there, working with me.’* (Participant 8)

The staff was characterized as genuinely interested, kind, empathic, and professional. Women described care during labor as respectful and warm. They frequently used the adjective ‘really’ to emphasize how they ‘really’ felt cared for. One woman said:

*‘… you can feel if a person really cares about you, really. That they really wish to make you feel better.’* (Participant 4)

Women reported feeling forever grateful and taking with them good memories for the rest of their lives. The quality of care was evident in the way they described the midwife as if she were a family member:

*‘For me it was like my mother or grandmother stood by my side. Like that. And it was easy to feel good. It was like she is not going to leave me in any case. She will make things good any way. It was so good.’* (Participant 6)

Professionalism and kindness were apparent in physical care. Women described midwives who cleaned them up when they were pushing without it being an issue. Women frequently used the word attitudes to describe the midwives as being respectful. Midwives were reported as not being told off when they vomited or passed stool when pushing. This positive attitude calmed them and created trust.

### Good healthcare system and health facilities

Participants reported that care was less medicalized than in their country of origin. They mentioned fewer blood tests and fewer ultrasounds. Most women were glad to avoid unnecessary investigations. A couple expressed concern about the limited number of consultations and lack of opportunity to have additional consultations and tests. They pointed out that there was no possibility for negotiation to deviate from the general standard set-up and defined this as lacking an individual approach. Most women, however, expressed gratefulness towards their new country and believed that the healthcare system in Norway prioritizes women’s health and maternity care.

Women were surprised that the public health services really were free of cost. They were impressed that it was not necessary to bring bedlinen, towels, clothes and nappies or any other baby items, as well as personal hygienic items. Those who had given birth in Ukraine previously mentioned taking with them many bags:

*‘Childbirth is 100% free of charge here … here they have everything … you do not need to worry before birth that you have to pack big bags, that was what it was like for me. You had to pack many things for the whole stay.’* (Participant 3)

All participants highlighted that the maternity units were modern, well supplied, clean and even beautiful. Having a room for themselves felt like luxury. The facilities had a positive influence on them. They mentioned that the dimmed lights and bathtub were soothing. Women were excited about the different types of equipment to assist them in moving during labor. Those who spent time in the operating theatre were impressed with the number of staff and the modern technology surrounding them:

*‘You can see the expensive machines, and everything. That there are many nurses, and doctors.’* (Participant 2)

### Challenges of being a refugee

None of the participating women spoke enough Norwegian to be able to communicate with staff. Language limitations and their unfamiliarity with how the Norwegian healthcare system works, were barriers to accessing and navigating the services.

### Problems communicating

All of the participants reported challenges with communication, in particular during labor. They used different methods to communicate across the language barrier. While Google translate worked for communicating short, concrete messages such as ‘you need an operation’, it was not sufficient for conveying detailed information or providing support. Several women mentioned that an interpreter via mobile telephone communication was frustrating even absurd:

*‘… I could not hear the interpreter because there were 10 doctors in the room … I did not hear the interpreter at all, because she translated when I had stopped pushing.’* (Participant 8)

One woman, who had a doula on the phone, was positive about interpretation via the phone during labor.

Not being able to communicate led to anxiety. Women felt they could not explain their situation and did not understand what the staff was telling them. Women tried to read information out of staff’s facial expressions and the intonation in the Norwegian they heard. They appreciated non-verbal support in the form of a smile and touch. However, the desire to understand lasted throughout labor. As one woman said:

*‘The worry was that there was a kind of black hole in the communication in my case … I did not know how my birth was progressing. I lacked information. I missed being told that.’* (Participant 2)

Even when information was given this was not always understood. One woman explained that she did not understand why she needed an operation during labor when she had given birth vaginally twice before. She recalled:

*‘The doctor used Google translate on his phone and said that I needed an operation … but I did not understand why I had to be operated. They put a kind of sensor on the baby’s head to listen to her brain or something like that. And then they told me that I need to be operated. That the baby is very tired.’* (Participant 1)

### Barriers to accessing services

Participating women expressed difficulties in navigating and adjusting to the Norwegian public health system. They were uncertain how, when, and whom to contact, in particular when labor started. Having to call the hospital before arriving, rather than just showing up, felt like a significant barrier. Explaining the frequency and intensity of contractions over the phone was challenging, and being told to wait at home created fear of giving birth before reaching the hospital. This happened to one of the participants.

Several women were uncertain how they would get to the hospital once labor started. A couple of them had been told that they could not just ring for an ambulance. However, a taxi was too expensive. In addition, they were worried that they would give birth in a taxi:

*‘We did not have a car… I had to give birth. Thankfully, the ambulance came. We tried to think of other alternatives. We had no other option. We did not know so much. We did not speak the language.’* (Participant 4)

## DISCUSSION

In this first study in Norway exploring Ukrainian refugee women’s experience with maternity care, women felt it was essential to first explain the healthcare in their home country in order to provide a context for their experiences with maternity care in Norway. Public healthcare in Ukraine is not provided free of charge, which adds a financial burden to families. In addition, women with experience of maternity care in their home country gave examples of disrespectful care. In contrast, they experienced the care in Norway as being of high quality, both staff and facilities. Women did struggle with the challenges of not speaking the language in their host country and unfamiliarity with the system, which made accessing and navigating services difficult.

Like the findings of a large qualitative study including migrants to Canada, the participants in our study contrasted their experiences in the host country to those of their country of origin^19^. In our study, women expressed satisfaction with their maternity care experiences, despite facing challenges. A similar pattern of satisfaction framed by comparison to care in their home country was found in a qualitative study from Finland including three Russian/Chechen participants^20^. This finding is in contrast to the overall conclusion of a systematic integrative review on humanitarian migrant women’s experiences of maternity care in Nordic countries, which reported feelings of insecurity, discrimination and diminished negotiation power^21^. However, most studies in this review included migrants from non-European countries with very different backgrounds to the women in our study^21^. A possible explanation for these contrasting findings can be that the Ukrainian women in our study had a similar physical appearance to Norwegian women, were familiar with a similar level of healthcare in relation to the use of technology and investigations, were well-educated, and experienced economic and social security due to the collective protection scheme they were admitted in.

The women in our study appreciated free maternity healthcare. This was a really important issue for them. Except for studies which provide evidence on the economic challenges of undocumented migrants in accessing healthcare, we found no studies reporting explicitly on the positive experience of free public healthcare^22^. It seems this aspect of care may be taken for granted or of less importance than other elements which contribute to the overall experience of maternity care. Norway has limited private practice options. It is possible to negotiate and pay for extra tests and ultrasound during pregnancy. During labor the only extra care that can be bought is the support of a doula. However, a doula who speaks Ukrainian/Russian is only available through publicly organized services in a limited number of communities in Norway. Research suggests that newly arrived migrant women with limited language skills and network, benefit greatly from being allocated a doula who understands their culture and language and can support them during pregnancy, early labor and accompany them in labor^23-25^. None of the women in our study had a doula physically with them during labor but one received support from a doula via the telephone. Doulas are not qualified interpreters, but their knowledge of midwifery and obstetric care and terminology allows them to improve understanding^23,25^. As illustrated in our study by the one woman in our study who received doula support over the telephone. She experienced this as beneficial in contrast to the woman who had a qualified interpreter on the phone. Indicating that a doula provides very different support than an interpreter.

Respectful care is now an integrated part of the guidelines for care in labor for a positive childbirth experience by WHO^12^. The literature on respectful and disrespectful maternity care has grown exponentially in recent years^26^, yet we found no studies from either Ukraine or Norway. Several meta-syntheses of qualitative studies present overlapping domains of what is the essence of respectful and disrespectful care^27,28^. Disrespectful care includes physical, emotional and verbal abuse; lack of information, choice, and consent; and discrimination^28,29^. Ukrainian women overall were positive about their childbirth experience in Norway, despite experiencing disrespectful care in the form of limited information, choice and informed consent. Both the perceived genuine care by the staff and the women’s expectation may have mitigated the negative effect of lack of information, choice and consent. None of the women reported experiencing discrimination. They did highlight being treated with dignity and respect in contrast to their experience in their country of origin. The literature on disrespectful care suggests that poorly organized care, poorly treated and renumerated staff, insufficient resources in the form of staff, equipment, medications, and space contribute to demotivated staff who treat patients with disrespect^29^. This may be the case for some of the public healthcare in Ukraine and result in the treatment described by the women in our study. Women highlighted how well equipped and staffed the maternity ward in Norway appeared to them.

The Norwegian Act on patient user rights ensures users of the healthcare services rights in regard to information, shared decision making and access^30^. For persons with inadequate Norwegian skills all public services are obliged to provide interpreter services^31^. These services may not be the most appropriate or easy to plan for use in spontaneous active labor. However, our findings indicate that interpretive services could be used more frequently during antenatal care so that women would know how the system works. The need for improved information about the services during pregnancy and increased use of interpreter services to reduce the extent of these barriers has been extensively recognized in previous research^32-35^.

### Strengths and limitations

A strength of the study was that the participants could express themselves freely because of the language skills of one of the authors. A common language and cultural understanding contributed to an atmosphere of trust during the interview and the richness of the data. A possible weakness of our study is the use of social media for recruitment and the inclusion criterion of speaking Russian fluently. However, most young women are on digital platforms, and many Ukrainians speak Russian. Most participants were well educated, which may increase their ability to find information. Participants were at varying stages of cross-cultural adaptation to life in Norway. Despite the aforementioned limitations we consider our findings transferable to other Ukrainian newly arrived refugees giving birth in Europe^36^

## CONCLUSIONS

Women compared the care received in Norway to the care in their country of origin. Overall, they perceived the care in their new country respectful and of high quality. The genuine care provided by staff during labor and efforts made to communicate, mitigated some of the negative effects of insufficient information and lack of participation in decisions during childbirth. Future research could investigate the benefit of language specific brochures on maternity services, include a list of commonly used specific childbirth terms in the languages of the home and the host country.

## Supplementary Material



## Data Availability

The data supporting this research are available from the authors on reasonable request.

## References

[1] European Council. Refugees from Ukraine in the EU. Accessed January 29, 2025. https://www.consilium.europa.eu/en/infographics/ukraine-refugees-eu/

[2] Statistisk sentralbyrå. Ukrainere er nå største flyktninggruppe i Norge. Accessed January 29, 2025. https://www.ssb.no/befolkning/innvandrere/statistikk/personer-med-flyktningbakgrunn/artikler/ukrainere-er-na-storste-flyktninggruppe-i-norge

[3] Harakow HI, Hvidman L, Wejse C, Eiset AH. Pregnancy complications among refugee women: a systematic review. Acta Obstet Gynecol Scand. 2021;100(4):649-657. doi:10.1111/aogs.14070 33372265

[4] Gieles NC, Tankink JB, van Midde M, et al. Maternal and perinatal outcomes of asylum seekers and undocumented migrants in Europe: a systematic review. Eur J Public Health. 2019;29(4):714-723. doi:10.1093/eurpub/ckz042 31098629 PMC6734941

[5] Verschuuren AEH, Tankink JB, Postma IR, et al. Suboptimal factors in maternal and newborn care for refugees: lessons learned from perinatal audits in the Netherlands. PLoS One. 2024;19(6):e0305764. doi:10.1371/journal.pone.0305764 38935661 PMC11210813

[6] Chrzan-Dętkoś M, Rodríguez-Muñoz MF, Krupelnytska L, et al. Good practices in perinatal mental health for women during wars and migrations: a narrative synthesis from the COST Action Riseup-PPD in the context of the war in Ukraine. Clín Salud. 2022;33(3):127-135. doi:10.5093/clysa2022a14

[7] Vallejo-Martín M, Sánchez Sancha A, Canto JM. Refugee women with a history of trauma: gender vulnerability in relation to post-traumatic stress disorder. Int J Environ Res Public Health. 2021;18(9):4806. doi:10.3390/ijerph18094806 33946312 PMC8125581

[8] Dempsey M, Peeren S. Keeping things under control: exploring migrant Eastern European women’s experiences of pregnancy in Ireland. J Reprod Infant Psychol. 2016;34(4):370-382. doi:10.1080/02646838.2016.1175552

[9] Kordan B. Russia’s war against Ukraine: historical narratives, geopolitics, and peace. Can Slavonic Pap. 2022;64(2-3):162-172. doi:10.1080/00085006.2022.2107835

[10] Garcia-Tizon Larroca S, Arevalo-Serrano J, Duran Vila A, et al. Human Development Index (HDI) of the maternal country of origin as a predictor of perinatal outcomes - a longitudinal study conducted in Spain. BMC Pregnancy Childbirth. 2017;17(1):314. doi:10.1186/s12884-017-1515-1 28934940 PMC5609033

[11] Sjetne IS, Iversen HH. Do experiences with pregnancy, birth and postnatal care in Norway vary by the women’s geographic origin? a comparison of cross-sectional survey results. BMC Pregnancy Childbirth. 2017;17(1):37. doi:10.1186/s12884-016-1214-3 28100175 PMC5241967

[12] World Health Organization. WHO recommendations: intrapartum care for a positive childbirth experience. WHO; 2018. Accessed January 29, 2025. https://www.who.int/publications/i/item/9789241550215 30070803

[13] Renfrew MJ, McFadden A, Bastos MH, et al. Midwifery and quality care: findings from a new evidence-informed framework for maternal and newborn care. Lancet. 2014;384(9948):1129-1145. doi:10.1016/S0140-6736(14)60789-3 24965816

[14] Fair F, Raben L, Watson H, et al. Migrant women’s experiences of pregnancy, childbirth and maternity care in European countries: a systematic review. PLoS One. 2020;15(2):e0228378. doi:10.1371/journal.pone.0228378 32045416 PMC7012401

[15] Węgrzynowska M, Sahraoui N, Nenko I, Szlendak B, Baranowska B. Ukrainian women’s maternity care strategies in Poland after the outbreak of the full-scale war: understanding unequal access to quality care. Soc Sci Med. 2024;362:117409. doi:10.1016/j.socscimed.2024.117409 39418931

[16] Chrzan-Dętkoś M, Murawska NE. “We are in this together” – Polish midwives’ reflections on perinatal care for Ukrainian women after the outbreak of war. Health Psychol Rep. 2023;11(3):177-187. doi:10.5114/hpr/161996 38084265 PMC10670767

[17] Regjeringen. Helseforskningsloven: Lov om medisinsk og helsefaglig forskning. 2022. Accessed January 29, 2025. https://www.regjeringen.no/no/dokumenter/helseforskningsloven/id2904358/

[18] Braun V, Clarke V. Toward good practice in thematic analysis: avoiding common problems and be(com) ing a knowing researcher. Int J Transgend Health. 2022;24(1):1-6. doi:10.1080/26895269.2022.2129597 36713144 PMC9879167

[19] Quintanilha M, Mayan MJ, Thompson J, Bell RC. Contrasting “back home” and “here”: How Northeast African migrant women perceive and experience health during pregnancy and postpartum in Canada. Int J Equity Health. 2016;15:80. doi:10.1186/s12939-016-0369-x 27225663 PMC4881207

[20] Lillrank A. Trust, vacillation, and neglect: refugee women’s experiences regarding pregnancy and birth giving in Finland. Nord J Migr Res. 2015;5(2):83-90. doi:10.1515/njmr-2015-0009

[21] Leppälä S, Lamminpää R, Gissler M, Vehviläinen-Julkunen K. Humanitarian migrant women’s experiences of maternity care in Nordic countries: a systematic integrative review of qualitative research. Midwifery. 2020;80:102572. doi:10.1016/j.midw.2019.102572 31739182

[22] Kisa S, Kisa A. “No Papers, No Treatment”: a scoping review of challenges faced by undocumented immigrants in accessing emergency healthcare. Int J Equity Health. 2024;23(1):184. doi:10.1186/s12939-024-02270-9 39277719 PMC11401389

[23] Erga-Johansen H, Bondas T. Multicultural doula care from the perspectives of immigrant women in Norway: a qualitative study. Sex Reprod Healthc. 2023;35:100827. doi:10.1016/j.srhc.2023.100827 36822024

[24] Bohren MA, Hofmeyr GJ, Sakala C, Fukuzawa RK, Cuthbert A. Continuous support for women during childbirth. Cochrane Database Syst Rev. 2017;7(7):CD003766. doi:10.1002/14651858.CD003766.pub6 28681500 PMC6483123

[25] Haugaard A, Tvedte SL, Severinsen MS, Henriksen L. Norwegian multicultural doulas’ experiences of supporting newly-arrived migrant women during pregnancy and childbirth: a qualitative study. Sex Reprod Healthc. 2020;26:100540. doi:10.1016/j.srhc.2020.100540 32622149

[26] Cantor AG, Jungbauer RM, Skelly AC, et al. Respectful maternity care: a systematic review. Ann Intern Med. 2024;177(1):50-64. doi:10.7326/M23-2676 38163377

[27] Shakibazadeh E, Namadian M, Bohren MA, et al. Respectful care during childbirth in health facilities globally: a qualitative evidence synthesis. BJOG. 2018;125(8):932-942. doi:10.1111/1471-0528.15015 29117644 PMC6033006

[28] Bohren MA, Berger BO, Munthe-Kaas H, Tunçalp Ö. Perceptions and experiences of labour companionship: a qualitative evidence synthesis. Cochrane Database Syst Rev. 2019;3(3):CD012449. doi:10.1002/14651858.CD012449.pub2 30883666 PMC6422112

[29] Bohren MA, Vogel JP, Hunter EC, et al. The mistreatment of women during childbirth in health facilities globally: a mixed-methods systematic review. PLoS Med. 2015;12(6):e1001847. doi:10.1371/journal.pmed.1001847 26126110 PMC4488322

[30] World Health Organization. Pasient- og brukerrettighetsloven, LOV 1999-07-02 nr 63 (Patient Rights’ Act). Accessed January 29, 2025. https://extranet.who.int/mindbank/item/1477

[31] Regjeringen. NOU 2014: 8 Interpreting in the public sector – a question relating to the right to due process of law and equal treatment. Accessed January 29, 2025. https://www.regjeringen.no/contentassets/a47e34bc4d7344a18192e28ce8b95b7b/no/sved/nou_2014_8_sammendrag_engelsk.pdf

[32] Bains S, Skråning S, Sundby J, Vangen S, Sørbye IK, Lindskog BV. Challenges and barriers to optimal maternity care for recently migrated women - a mixed-method study in Norway. BMC Pregnancy Childbirth. 2021;21(1):686. doi:10.1186/s12884-021-04131-7 34620114 PMC8495671

[33] Leppälä S, Lamminpää R, Gissler M, Vehviläinen-Julkunen K. Hindrances and facilitators in humanitarian migrants’ maternity care in Finland: qualitative study applying the three delays model framework. Scand J Caring Sci. 2020;34(1):148-156. doi:10.1111/scs.12716 31149746

[34] Ternström E, Small R, Lindgren H. Migrant women’s experiences of an individual language-assisted information and support visit to the labor ward before giving birth - A qualitative study from Sweden. Sex Reprod Healthc. 2023;38:100915. doi:10.1016/j.srhc.2023.100915 37717410

[35] Akselsson A, Westholm L, Small R, Ternström E. Midwives’ communication with non-Swedish-speaking women giving birth: a survey from a multicultural setting in Sweden. Eur J Midwifery. 2022;6:38. doi:10.18332/ejm/148159 35801227 PMC9201782

[36] Graneheim UH, Lindgren BM, Lundman B. Methodological challenges in qualitative content analysis: a discussion paper. Nurse Educ Today. 2017;56:29-34. doi:10.1016/j.nedt.2017.06.002 28651100

