# Supplementary material for: Ukrainian refugee women’s experience with maternity care in Norway: A qualitative study
Source: Eur J Midwifery. 2025 Feb 12;9:10.18332/ejm/200613. doi: 10.18332/ejm/200613 (PMC11815948; doi:10.18332/ejm/200613)
Supplement: Supplementary file 1 [file EJM-9-10-s1.pdf]

Supplementary file 1

Interview guide for the first two interviews

1. Please tell med about your childbirth experience in Norway?

2. What expectations did you have about giving birth and what do you think now?

3. What did you know about maternity care in Norway before giving birth?

4. How would you describe your meeting with the midwife (midwives) and the care they provided?

5. Can you recall some central moments/things that were challenging or annoying?

6. Can you recall some central moments/things that were positive?

To ensure more information on the experience with midwives this question was moved to the start of the interview. The question about important moments in care consisted first of two questions, one for positive experiences and one for negative experiences and was joined into one question.

Interview guide after minor changes

1. Please tell me about your childbirth experience in Norway?

2. How would you describe your meeting with the midwife (midwives) and the care they provided?

3. What expectations did you have about giving birth and what do you think now?

4. What did you know about maternity care in Norway before giving birth?

5. Can you recall some central moments/things that were challenging or annoying? Any moments/things you remember as positive?

©2025 Lukasse M. et al.
